# Thermodynamic Modelling and Microstructural Study of Z-Phase Formation in a Ta-Alloyed Martensitic Steel

**DOI:** 10.3390/ma14061332

**Published:** 2021-03-10

**Authors:** Florian Riedlsperger, Bernadette Gsellmann, Erwin Povoden-Karadeniz, Oriana Tassa, Susanna Matera, Mária Dománková, Florian Kauffmann, Ernst Kozeschnik, Bernhard Sonderegger

**Affiliations:** 1 Institute of Materials Science, Joining and Forming (IMAT), Graz University of Technology, 8010 Graz, Austria; bernadette.gsellmann@gmx.at; 2 Christian-Doppler Laboratory for Interfaces and Precipitation Engineering CDL-IPE, TU Wien, 1060 Wien, Austria; erwin.povoden-karadeniz@tuwien.ac.at; 3 Institute of Materials Science and Technology, TU Wien, 1060 Wien, Austria; ernst.kozeschnik@tuwien.ac.at; 4 CSM Centro Sviluppo Materiali, 00128 Rome, Italy; oriana.tassa@rina.org (O.T.); susanna.matera@rina.org (S.M.); 5 Institute of Materials Science of MTF STU, 917 24 Trnava, Slovakia; maria.domankova@stuba.sk; 6 Materials Testing Institute (MPA), University of Stuttgart, 70569 Stuttgart, Germany; florian.kauffmann@mpa.uni-stuttgart.de (F.K.); bernhard.sonderegger@jku.at (B.S.); 7 Institut für Metallische Konstruktionswerkstoffe, JKU Linz, 4040 Linz, Austria

**Keywords:** thermodynamics, precipitate kinetics, microstructure, modelling, steel, creep

## Abstract

A thermokinetic computational framework for precipitate transformation simulations in Ta-containing martensitic Z-steels was developed, including Calphad thermodynamics, diffusion mobility data from the literature, and a kinetic parameter setup that considered precipitation sites, interfacial energies and dislocation density evolution. The thermodynamics of Ta-containing subsystems were assessed by atomic solubility data and enthalpies from the literature as well as from the experimental dissolution temperature of Ta-based Z-phase CrTaN obtained from differential scanning calorimetry. Accompanied by a comprehensive transmission electron microscopy analysis of the microstructure, thermokinetic precipitation simulations with a wide-ranging and well-documented set of input parameters were carried out in MatCalc for one sample alloy. A special focus was placed on modelling the transformation of MX into the Z-phase, which was driven by Cr diffusion. The simulation results showed excellent agreement with experimental data in regard to size, number density and chemical composition of the precipitates, showing the usability of the developed thermokinetic simulation framework.

## 1. Introduction

Martensitic 9–12% Cr steels are widely used high-temperature alloys with excellent stability against corrosion, outstanding creep resistance [1] and low manufacturing costs [2] compared to austenitic steels or nickel base alloys. They are reasonably tough, can resist frequent thermal loads [3] and combine good thermal conductivity with low thermal expansion [4]. The main fields of application for steel grades such as E911, P91 and P92 today are in turbine parts or surroundings, pipes and boilers or heat exchangers in thermal power plants [5]. In order to reduce the CO_2_ emissions of fossil-fired power plants, their thermal efficiency must be further maximized by increasing the operational temperature and pressure [6]. In fact, elevated temperature environments pose a challenge in terms of creep resistance. Steels with 9–12% Cr owe their high creep strength to a fine sub-structure that is embedded in martensitic packets and blocks inside of prior austenitic grains and to numerous precipitates located at grain and subgrain boundaries [7]. Apart from the chromium-carbide M_23_C_6_, MX carbonitrides in particular provide significant precipitate strengthening [8] due to their small size and high number density. These precipitates both impede mobile dislocation movement and are assumed to pin subgrain boundaries [8,9,10]. Traditionally, finely distributed MX carbonitrides, typically in the size range of 20 to 40 nm [11,12], have been introduced by the addition of V and Nb (e.g., in P91 and P92). However, the optimized precipitate distribution degrades within the scope of MX transformation into thermodynamically more favourable, coarse modified Z-phase in the order of hundreds of nm [13] by Cr in-diffusion [14]. MX transformation into modified Z-phase is made responsible for a breakdown in creep life and premature failure [15].

Two strategies have been proposed to avoid these issues: (i) Formation of Z-phase can be slowed down and its detrimental effect may be delayed as long as possible—ideally beyond the planned service life of power plant components—by alloying adjustment (e.g., avoiding high Cr contents, such as in P122) [6]. (ii) Development of Z-steels: a new promising alternative is to replace V and Nb in MX carbonitrides by Ta [16] in 11–12% Cr steels. Ta(C,N) transforms very quickly into small and evenly distributed CrTaN Z-phase particles already during heat treatments in the manufacturing process [17]. Strikingly, this Ta-based Z-phase turned out to have high stability against coarsening during creep [18,19].

For modified Z-phase of the type Cr(V,Nb)N, four formation mechanisms are pro-posed [20]: (i.) already existing face-centred cubic (fcc) MX carbonitride precursors serve as nucleation sites, or (ii.) MX transforms into Z-phase, supported by Cr-consumption from the matrix [20]; it has also been observed that (iii.) hexagonal closed packed (hcp) M_2_X precursors (of the composition Cr_2_N) may act as spots of Z-phase nucleation [21], respectively, and (iv.) may transform into Z-phase due to V/Nb intake. 

Similar processes take place in Z-steels: Cr entering into MX or Ta diffusing into M_2_X have been shown as (trans)formation mechanisms for Ta-based Z-phase [22].

From a crystallographic point of view, fcc MX or hcp M_2_X precursors are replaced by cubic and later tetragonal Z-phase [14,23] to minimize the Gibbs energy and reach thermodynamic stability. The authors of [23] interpret the cubic structure as a metastable, transient Z-phase stage, whereas those of [16] propose the co-existence of cubic and tetragonal structures.

For technical usability of Z-steels, MX precursors are generally preferred, since they result in small Z-phases, whereas the M_2_X mechanism may produce undesired oversized Z-phases [22]. Three factors control the question of which precursor type predominates: (i.) heat-treatment [21,22] (particularly the tempering conditions), (ii.) C content [22] (the higher, the more MX) and (iii.) Ta/N ratio (the more excess N, the more M_2_X) [22].

A special characteristic of Ta-based Z-phase is its stoichiometry of Cr_1+x_Ta_1-x_N with x = 0.2 in final stage [24]. To determine the degree of transformation, often the Cr:Ta ratio is stated [25]. For a fully evolved Ta-based Z-phase with completed element intake, Cr:Ta = 1.5 [25]. In contrast, so-called MX/Z-hybrid particles define an intermediate stage with Cr:Ta = 0.5, indicating that the transformation has started or is currently ongoing [26]. In-depth microstructural analysis revealed that they may contain zones of both MX and Z-phase [14].

In this work, we present the first study of Calphad thermodynamics-based predic­tions of precipitate evolution in a Ta-containing Z-steel. Results of the thermokinetic pre­cipitation simulation with MatCalc [27] are validated by transmission electron microscopy (TEM) results and atom probe tomography (APT) data from the literature [18]. A thermodynamic description of Ta-based Z-phase was ascertained by differential scanning calorimetry (DSC) and implemented in the open-source steel database “mc_fe” [28].

## 2. Materials and Methods

Two 10–12% Cr-steels, denoted as ZULC (ultra-low carbon) and Z6, were investi­gated. ZULC was one of the first trial steels to investigate the characteristics of Ta-based Z-phase; the results have been well documented [18,19,25]. ZULC in this work served to detect the dissolution temperature of the Z-phase. Z6 ranks amongst the latest candidates for industrial application of this material group and has been recently developed within the EU project CRESTA2 (RFSR-CT-2014-00032). For Z6, a detailed microstructural and phase analysis was conducted in this work along with equilibrium and thermokinetic simulations.

**Chemical compositions** of ZULC and Z6 are shown in Table 1. The main differences of these concern C content (higher in Z6) and Cr (more in ZULC). The Ta and N contents are very similar. The chemical composition of Z6 was measured in this work by optical emission spectrometry (OEM), except for Ta content, which had been previously determined by X-ray fluorescence spectroscopy (XRF) [29]. Please note that the C-content of Z6 from OEM (0.017 wt.%) significantly differed from the value documented in [29] (only 0.009 wt.%) which was obtained by combustion analysis of the melt. Chemistry of ZULC was adopted from the literature [18,19,25].

**Simulation**. Thermodynamic equilibrium and precipitate kinetic simulations were run by MatCalc version 6.03 (rel. 1.003) on a Windows 10 system with 64 bits and 4 Intel Core 2 Quad Q9550 @ 2.83 GHz processors. The used databases were “mc_fe_v2.061.tdb” (for thermodynamics) and “mc_fe_v2.013.ddb” (for diffusion data). Only equilibrium was calculated for ZULC. Equilibrium and precipitate kinetics—consisting of the stages of normalizing, tempering and ageing—were simulated for Z6.

**Microstructural investigations and phase characterization**. Basic microstructural and phase information such as grain and subgrain sizes, but also sites of preferred precipitate nucleation, represent important inputs for the thermokinetic simulation setup. On the other hand, experimental observations allow plausibility checks of the simulation results and provide a deepened insight into precipitate characteristics, i.e., particle sizes, number densities and compositions. 

The TEM analysis findings were employed for Z6. A FEG JEOL 3200FS operated at 300 kV from CSM (Centro Sviluppo Materiali) was used for the TEM precipitate analysis. Carbon extraction replicas were prepared as follows: Samples were polished and Vilella etched at 20 °C (without electrochemical device). Samples were then cleaned before evaporating carbon on them. Finally, the replicas were stripped with a geometry of 2 × 2 mm^2^, etched again (making stripped areas peel off) and applied to a TEM copper grid. Beam conditions in selected area diffraction (SAD) were chosen for generating dark and bright field diffraction contrast images. Precipitate phases were identified by crystallographic analysis with SAD and by energy-dispersive X-ray spectroscopy (EDX).

The TEM measurement of dislocations and subgrains in Z6 was performed at MTF STU (Slovak University of Technology). For this purpose, thin foils were prepared. Discs with a diameter of 3 mm and a thickness of 100 µm were jet-electropolished in electrolyte HNO_3_:CH_3_OH = 3:7 at 0 °C and 15 V to obtain transparent areas near the central hole. Bright-field images were produced from a microscope JEOL 200CX operating at 200 kV. A mean linear intercept method was used to quantify observed dislocations in subgrain interiors (in the following referred to as *ρ*_int_): An array of horizontal and vertical lines was introduced to the TEM images and intersection points were counted. The number of intersections was then weighted according to line length and sample thickness to obtain dislocation densities. For further details on the method, see, e.g., [12].

The chemistry of Z6 was confirmed by OES at MPA Stuttgart, applying 4 sparks to a 21 × 21 × 10 mm^3^ sample in condition 0 (before the start of normalizing).

An overview of all investigated Z6 sample conditions is given in Section 5.

The dissolution temperature of Ta-based Z-phase was determined by DSC at TU Wien on a normalized ZULC sample provided by Chalmers University of Technology. Tests were run on a high-temperature DSC machine 404C Pegasus (manufactured by the company Netzsch in Selb, Germany) at 20 K/min heating and cooling.

## 3. Incorporation of Ta into Thermodynamic and Diffusion Mobility Databases

For simulations of the transformation from MX to Z-phase in Ta-alloyed Cr-steel, multicomponent thermodynamic and diffusion mobilities in “mc-fe” databases (open-license databases “mc_fe_v2.060.tdb” and “mc_fe_v2.012.ddb” [28]) were extended by thermodynamic information on Ta. Key parameters in this regard are the solubilities of Ta in MX, M_2_X, and Z-phase. Associated solubility limits of Ta in austenitic and ferritic alloy phases play an essential role as well. Interaction energies between Fe and Ta, and Cr and Ta in ferrite and austenite were taken from the thermodynamic Calphad description of binary Fe-Ta [30] and Cr-Ta [31], respectively. In “mc_fe_v2.060”, TaC and TaN compound energies were added to the description of fcc-type MX carbonitrides, based on available Ta-C, Ta-N Calphad descriptions and considerations of Ta-C-N phase stabilities [32,33]. Recently analyzed first principles of TaN formation enthalpies by Grumski et al. [34] were preferred in this study over Li et al. [35] and were considered in the model parametrization of Ta-MX.

Hcp Cr_2_N-type carbonitride (M_2_X) precipitates were identified as relevant “competi­tors” to MX as Z-phase precursor phases [22]. This especially applies for Z-steels with low C and high N content [22]. Moreover, significant Cr-content in MX has been found [18,22]. Thus, in addition to binary and ternary Ta-C and Ta-C-N stability considerations, Cr-solubilities in respective carbonitrides needed to be described by appropriate interaction parameters among Cr and Ta. This enabled us to modulate the Gibbs energies of mixed MX (Cr,Ta)(N,C) and M_2_X (Cr,Ta)_2_(C,N).

The thermodynamic description of Z-phase nitride is defined by the compound energy sublattice formula in analogy to Danielsen and Hald [36], (Cr)(Ta)(N,Va), i.e., replacing Nb and V in the second sublattice by Ta. Va denotes empty sites in the interstitial sublattice. The full thermodynamic sublattice description for combined Ta-containing Z-phase and Cr(V,Nb)N Z-phase, implemented in the “mc_fe” database, (Cr,Fe)(Nb,Mo,Ta,V)(N,Va), allows for the replacement of Nb, V and Mo in modified Z-phase by Ta. Since the equilibrium stoichiometry of Z-phase was found to be Cr_1.2_Ta_0.8_N [24] rather than CrTaN, the solubility of Cr was allowed also on the second sublattice. Subsequently, this led to a final sublattice description of (Cr,Fe)(Cr,Nb,Mo,Ta,V)(N,Va). The compound energy of metastable (Cr)(Cr)(N) and the interaction energy between Cr and Ta in the second sublattice, (Cr)(Cr,Ta)(N), was adjusted in order to reproduce the experimental Z-phase stoichiometry. The compound enthalpy is of prime importance for the thermodynamic stability of Z-phase and also for the interfacial energy in precipitation simulations, see, e.g., [37]. In the present thermodynamic description, the enthalpy is approximated to the first-principles result of Urban and Elsässer [38]. The entropic contribution to the molar Gibbs energy of the Z-phase compound (Cr)(Ta)(N) was then adapted to the temperature limit of Z-phase stability, as determined by DSC. Moreover, vacancies were assumed to replace part of the nitrogen, in agreement with previous thermodynamic modelling of Nb-V-Z (Danielsen and Hald [36]). The resulting calculated molar entropy of Z-phase was close to the values of fcc and hcp Cr-Ta nitrides.

The relative phase stabilities of MX and Z-phase obey the criterion of metastable MX [39], which transforms into Z-phase when equilibrium conditions are approached, i.e., after long-term annealing.

Table 2 provides an overview of all the thermodynamic interaction parameters and revised sublattice descriptions added to the “mc_fe.tdb” database. Elements with % are major constituents.

Proposed diffusion mobilities of Ta in ferrite and austenite (taken from Fridberg [40]) were inserted into “mc_fe.ddb”.

The extended MatCalc databases provided us with the opportunity to simulate Ta-containing alloys. Input parameters are needed for the simulations in MatCalc, which are specified in the following.

## 4. Input Parameters for Thermokinetic Simulations

MatCalc employs the classical nucleation theory (CNT) [41] and calculates growth and coarsening of precipitates as a side product of the SFFK (Svoboda–Fischer–Fratzl–Kozeschnik) model [42], which is based on Onsager’s extremum principle [43]. SFFK takes into account the energy dissipation from a) diffusion in the matrix, b) diffusion in the precipitates and c) moving interfaces [42]. In addition, interfacial energies are calculated automatically by MatCalc; for the theory, please refer to the generalized broken-bond model (GBB) [37,44].

Before starting simulations of Ta-containing alloys in MatCalc by applying the new databases, input parameters have to be defined as follows.

### 4.1. Chemistry

The chemical composition is indicated in Table 1.

### 4.2. Simulated Precipitate Phases

Apart from MX, M_2_X and Z-phase, M_23_C_6_ chromium-carbide and the Laves phase (Fe,Cr)_2_(Mo,W) were also chosen as precipitate phases. Despite the fact that the focus of this study was on the kinetics of Z-phase precursors and their transformation to Z-phase, M_23_C_6_ and Laves phase need to be considered for appropriate interpretations of the simu­lation, since these precipitates contribute to the distribution of relevant elements, such as Cr and Mo during heat treatment. BN was indirectly taken into account in the simulations. The N content was reduced from nominal 0.035 wt.% to 0.031 wt.%, using the result of the equilibrium calculation, which predicted 0.004 wt.% N to be bound in BN. In this way, the impact of less available N on MX precursors and subsequently on the Z-phase was accounted for.

### 4.3. Simulation Modes

Equilibrium calculations between 500 and 1500 °C reveal thermodynamically stable phase fractions and require the chemical composition as the only input.

For precipitation kinetic simulations (this work: Z6), we need additional input parameters, including microstructural information and nucleation settings; see Section 4.4 to Section 4.8.

### 4.4. Nucleation Sites

In the context of nucleation sites (see Table 3), the austenitic matrix must be distinguished from the martensitic matrix. In terms of heterogeneous sites for nucleation, dislocations (d) are relevant in austenite. In martensite, grain boundaries (g) and subgrain boundaries (s) are considered in addition. For Z-phase, MX precursor carbonitrides serve as precipitation sites.

### 4.5. Z-Phase Transformation Model

For Z-phase precipitation on MX precursors, a direct particle transformation model with an equivalent interface energy was used, continuing an approach of [45]. The model reflects the nucleation process of a new phase, here Z-phase, occurring within the volume of precursor precipitates, here MX. This mechanism is denoted as inner-particle nuclea­tion. The transformation is only enabled, if the product phase (Z-phase) is thermodynam­ically more stable than the parent phase (MX); i.e., the stabilities of parent and product phase are continuously compared based on their modelled thermodynamic driving forces. The driving force difference of the two phases serves as effective driving force for nucle­ation of the product phase. This effective driving force (expressed per volume), $\Delta G_{0,\mathrm{eff}}$, together with an equivalent interface energy, $\gamma_{0,\mathrm{equ}}$, enters the critical nucleation energy, G*, and thus determines the nucleation rate, *J*, of the product phase (Equations (1) and (2)).

(1) $$J=N_{0}f_{Z}\beta^{*}\cdot exp\left\{-\frac{G^{*}}{k_{B}T}\right\}\cdot exp\left\{-\frac{\tau }{t}\right\}$$

(2) $$G^{*}=\frac{16\pi }{3}\cdot \frac{\gamma_{0,\mathrm{equ}}^{3}}{{\left(\Delta G_{0,\mathrm{eff}}\right)}^{2}}$$

The number of available nucleation sites for the product phase, *N*_0_, corresponds to the number of atoms on the surface of the parent precipitates. Apart from *N*_0_, Boltzmann constant *k*_B_, Zeldovich factor *f*_Z_, and atomic attachment rate $\beta^{*}$ are also contained in *J*. Whereas *f*_Z_ is calculated identically to the case of homogeneous nucleation—related to the probability of a critical nucleus to not dissolve again—$\beta^{*}$ is set to unity because of all elements being already present. The incubation time, *τ*, is a function of *f*_Z_ and $\beta^{*}$. Once a number of ∫*J*.d*t* precipitates of the product phase have nucleated (after time *t* and at a certain temperature *T*), an equal number of precursor precipitates is removed from the subsequent simulation increment [46]. For more details on transient nucleation in multi­component systems, see [47,48]. A similar model has recently been proposed for the phase transformation of carbides [49], lacking however an embedding into an advanced thermokinetic framework as offered by MatCalc.

### 4.6. Heat Treatment

**Figure 1:**
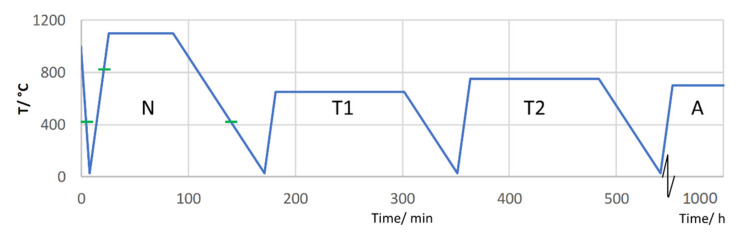
Thermal history of Z6.

Simulative heat treatment setup of Z6 comprised several steps: Z6 was first normalized (N) for 1 hour at 1100 °C. Double-step tempering for 2 h at 650 °C (T1) and for 2 h at 750 °C (T2) was finally followed by ageing (A) for 1000 h at 700 °C. The thermal history of Z6 is portrayed in Figure 1.

When exceeding the re-austenitization temperature of 820 °C during heating, MatCalc switches the precipitation domain from martensite to austenite. During cooling, 420 °C was defined as transition temperature from austenite to martensite matrix. Both matrix changes are symbolized in Figure 1 by horizontal green lines before and during N.

### 4.7. Microstructural Settings

Regarding the microstructure of Z6, a prior austenitic grain size (diameter) of 48 µm was implemented in accordance with experimental results [29]. The initial subgrain size (diameter) was set at 0.41 µm in agreement with TEM measurements—see Section 5.2.

Dislocation densities were varied throughout the simulation. For austenite, a dislocation density of 10^11^ m^-2^ was adopted from [45]. For martensite, the static recovery part of a state-parameter-based recrystallization model from [50,51] was implemented. The evolution of dislocation density $\rho_{\mathrm{int}}$ in subgrain interiors can subsequently be described by Equation (3):

(3) $${\dot{\rho }}_{\mathrm{int}}=-2CD_{s}\frac{Gb^{3}}{k_{B}T}\left(\rho_{\mathrm{int}}^{2}-\rho_{\mathrm{int},\mathrm{eq}}^{2}\right)$$

where *b* denotes the Burgers vector, *D*_s_ the self-diffusion coefficient (taken from [52]), *G* the shear modulus and *T* the temperature. $\rho_{\mathrm{int},\mathrm{eq}}$ is the equilibrium dislocation density. Parameter *C* controls the speed of static recovery [50].

For the normalizing and tempering, the dislocation density of fresh martensite was distinguished from that of tempered martensite based on TEM analysis. Between those two different levels of dislocation density, a simulation for static recovery was carried out, making use of Equation (3). Parameter *C* was set to 3 × 10^−4^. During ageing, static recovery was modelled in the same way, being supported by our TEM data.

### 4.8. Z-Phase Settings

For Z-phase precipitates and their nucleation linked to MX precursors, the transformation settings turned out to be essential, including an equivalent interface energy $\gamma_{0,\mathrm{equ}}$ of 0.22 Jm^−2^ and a minimum nucleation radius (*mnr*) of 5 Å. *mnr* was estimated based on crystallographic unit cell and lattice parameters of Z-phase as stated in [16,53]. For further discussion, please refer to Section 7.5.

Table 4 summarizes all named input parameters for the precipitation kinetic simulation of Z6, in addition to the already given chemical composition (Table 1) and nucleation sites (Table 3).

## 5. Results of the TEM Measurements

**Figure 2:**
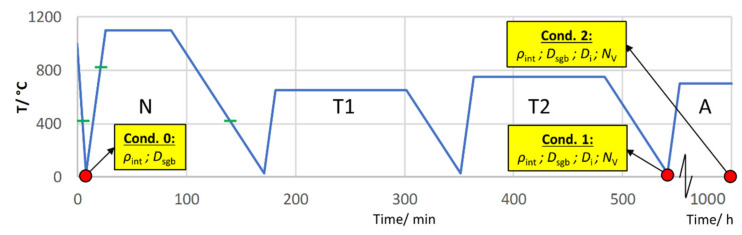
Investigated sample conditions and microstructural features of Z6.

Figure 2 portrays all investigated sample conditions (0, 1 and 2) and shows which microstructural features were characterized. *ρ*_int_ denotes the internal dislocation density, *D*_sgb_ the subgrain size, *D*_i_ the precipitate size and *N*_V_ the number density.

### 5.1. Precipitates

**Figure 3:**
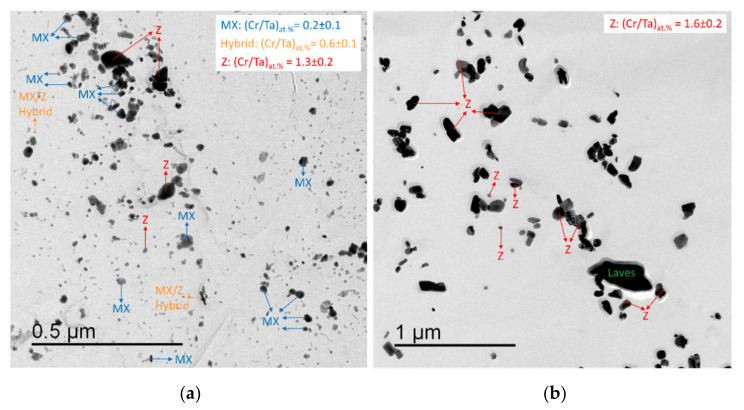
TEM extraction replicas of Z6 in (**a**) condition 1 and (**b**) condition 2.

Figure 3 shows TEM impressions of precipitates in (a) condition 1 (N + T) and (b) condition 2 (1000 h aged at 700 °C). The average Cr:Ta ratio is given for MX and Z-phases, which were also identified by SAD. MX/Z-hybrid particles [26] in the as-received state show that the transformation was currently ongoing and not yet completed. After ageing, all MX and MX/Z-hybrids appeared to be completely replaced by coarsened Z-phase precipitates. Apart from that, some large Laves phase and M_23_C_6_ precipitates close to and above the µm scale, respectively, were detected after ageing.

Table 5 summarizes the observed precipitate sizes *D*_i_ and the total number densities *N*_V_ in the two conditions obtained from extraction replica, which were investigated by CSM.

It must be mentioned that the number of investigated particles was very low for M_23_C_6_. In contrast, for the determination of MX and Z-phase sizes, around one hundred particles were analyzed and distinguished by EDX (*N*_EDX_). For evaluation of total number densities, several thousand (number *N*_Size_) were counted, but not individually characterized by EDX. In this context, analysis of the measured size distributions with support of our thermokinetic simulation in Section 6.3.1 and Section 6.3.2 (see Figures 11 and 14) will foster a deeper and statistically more profound insight into how the total number densities can be split into different precipitate populations.

### 5.2. Dislocation Densities and Subgrain Sizes

Internal dislocation density (*ρ*_int_) and the subgrain size (diameter *D*_sgb_) were determined by TEM.

**Figure 4:**
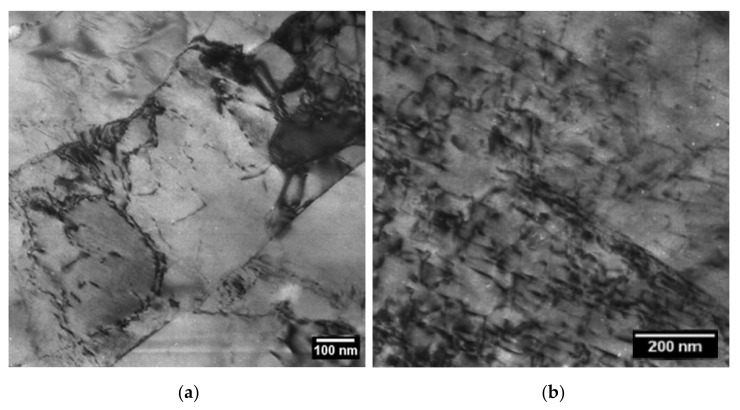
(**a**) Subgrain boundaries and (**b**) internal dislocations in condition 1 (N + T).

**Figure 5:**
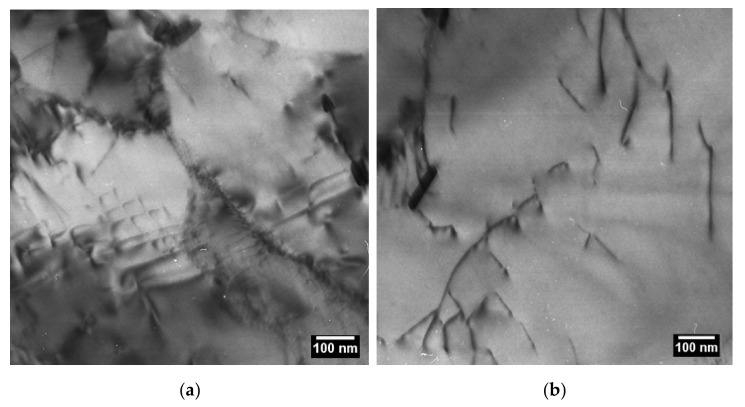
(**a**) Subgrain boundaries and (**b**) internal dislocations in condition 2 (1000 h, 700 °C).

To give an impression, Figure 4 shows (a) some subgrain boundaries and (b) a high number of internal dislocations in the material in as-received condition 1 (N + T). For comparison, Figure 5 displays the 1000 h aged condition 2 of (a) subgrains and (b) internal dislocations.

Table 6 provides an overview of all obtained results in conditions 0, 1 and 2.

Martensite before the start of normalizing (condition 0) turned out to have the highest dislocation density *ρ*_int_ at 6.0 × 10^14^ m^−2^. Strong static recovery occurred during tempering, producing tempered martensite with *ρ*_int_ = 2.6 × 10^14^ m^−2^ (condition 1). Reduction of dislocation density further continued during ageing, reaching *ρ*_int_ = 7.9 × 10^13^ m^−2^ after 1000 h of heat input at 700 °C (condition 2).

*D*_sgb_ turned out to be relatively similar before normalizing, in the as-received state (N + T) and in the aged condition (1000 h @ 700 °C), yielding values of 0.48, 0.41 and 0.43 µm.

## 6. Results of MatCalc Simulations

This chapter presents simulation results (data) from MatCalc which are also pro-vided as supplement part 2 of this work.

### 6.1. Equilibrium ZULC and Z6

**Figure 6:**
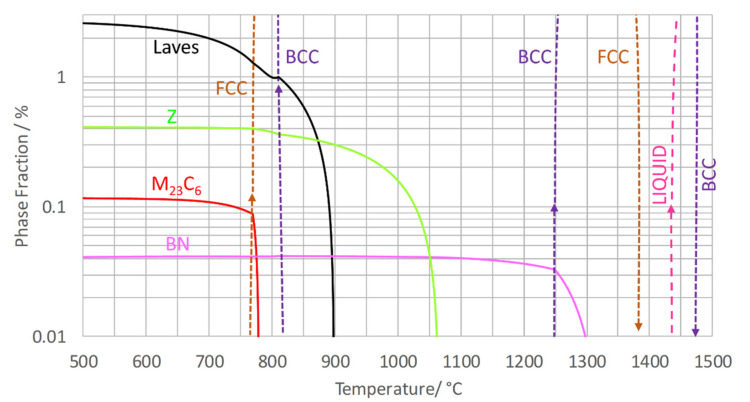
Calculated molar equilibrium phase fractions in ZULC.

**Figure 7:**
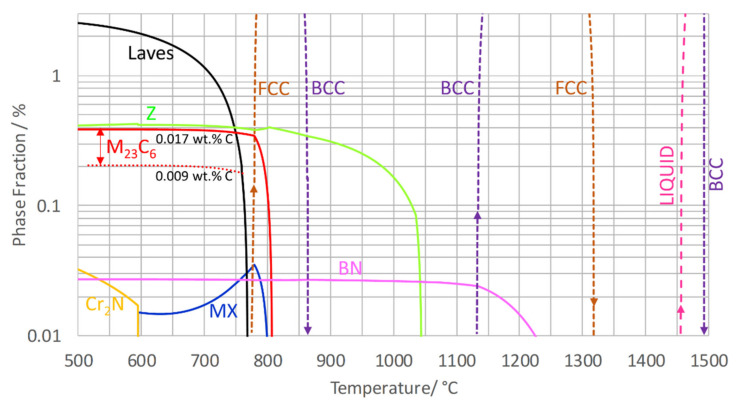
Calculated molar equilibrium phase fractions in Z6.

Equilibrium calculations of ZULC and Z6 show that between 550 °C and 700 °C (see Table 7; Figure 6 and Figure 7), the Laves phase had the highest computed molar phase fraction at 1.2 to 2.5%, followed by Z-phase at approximately 0.4%. M_23_C_6_ is predicted to exist in ZULC with a 0.1% phase fraction. In Z6, more M_23_C_6_ is anticipated with 0.2 to 0.4% due to a higher C content (0.009 to 0.017 wt.%). Traces of fcc MX (0.02%) become stable for Z6 in a range between 595 °C and 800 °C. Underneath the forming temperature of MX, 0.02 to 0.03% hcp Cr_2_N with negligible content of Ta is predicted in Z6. By contrast, neither MX nor Cr_2_N occurs in equilibrium of ZULC. The computed theoretical dissolution temperatures of Z-phase at 1065 °C (ZULC, Figure 6) and 1050 °C (Z6, Figure 7) agree with the exothermal DSC peak of the cooling experiment, assuming an undercooling correction of approximately 50 °C due to kinetic barriers for Z-phase precipitation. In Z6, the lower Z-phase dissolution temperature compared to ZULC is related to stable secondary MX par­ticles appearing between 1040 and 1070 °C. According to MatCalc, BN forms in both alloys with a phase fraction of 0.03 to 0.04% (more in ZULC due to the higher content of B).

### 6.2. Simulation of Dislocation Evolution

Before starting to simulate the precipitation kinetics of Z6, the evolution of dislocation density in martensite needs to be carefully modelled and implemented in MatCalc. This concerns the stages of tempering and ageing. Here, we present the results of the previously introduced dislocation evolution model (Equation (3)), linking the obtained TEM data points from Table 6.

**Figure 8:**
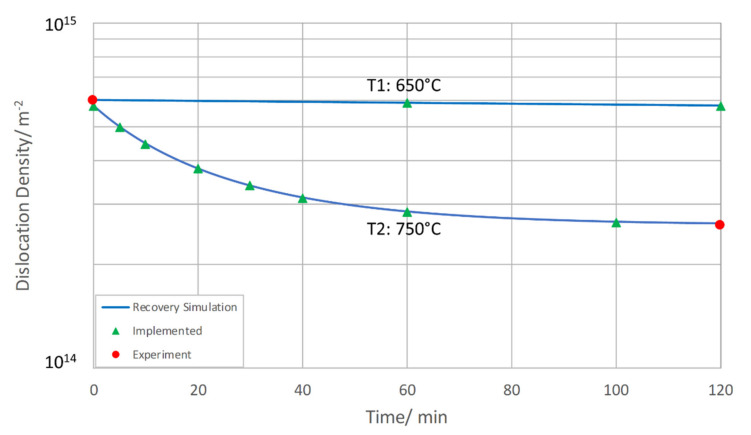
Simulated static recovery during tempering at 650 °C (T1) and 750 °C (T2).

During tempering, a simulation of static recovery was conducted using Equation (3). It turns out that the fastest recovery took place during the second tempering step at 750 °C (T2), whereas dislocation density remained almost unchanged during the first tempering step at 650 °C (T1). In Figure 8, the underlying TEM start value of 6 × 10^14^ m^−2^ and TEM end value of 2.6 × 10^14^ m^−2^ are marked by points, the recovery simulation is a full line and the implemented data in MatCalc (additional to the TEM start and end values) are outlined in triangles.

**Figure 9:**
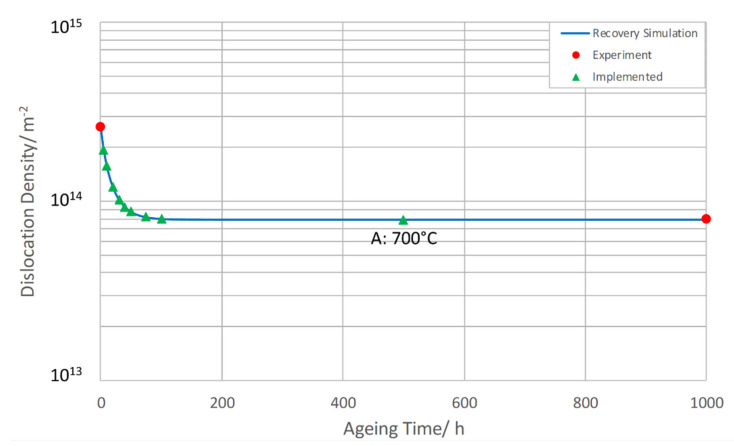
Simulated static recovery of dislocation density during ageing.

During ageing, static recovery was simulated in the same way, and was incorporated stepwise into MatCalc and calibrated again by our TEM data in as-received condition (N + T) as well as after 1000 h of ageing at 700 °C (see Figure 9).

### 6.3. Precipitate Kinetic Simulation

This section discusses the results of Z6 precipitation kinetic simulations and compares them to measured TEM data with respect to sizes and number densities. For the simulation shown here, a carbon content of 0.009 wt.% (obtained from combustion analysis) was chosen. In parallel, we also demonstrate and include the effect of a higher C-content (0.017 wt.% from OEM) on the evolution of number density and phase fraction of M_23_C_6_.

#### 6.3.1. Z6, Heat Treatment Up to Condition 1 (N + T)

**Figure 10:**
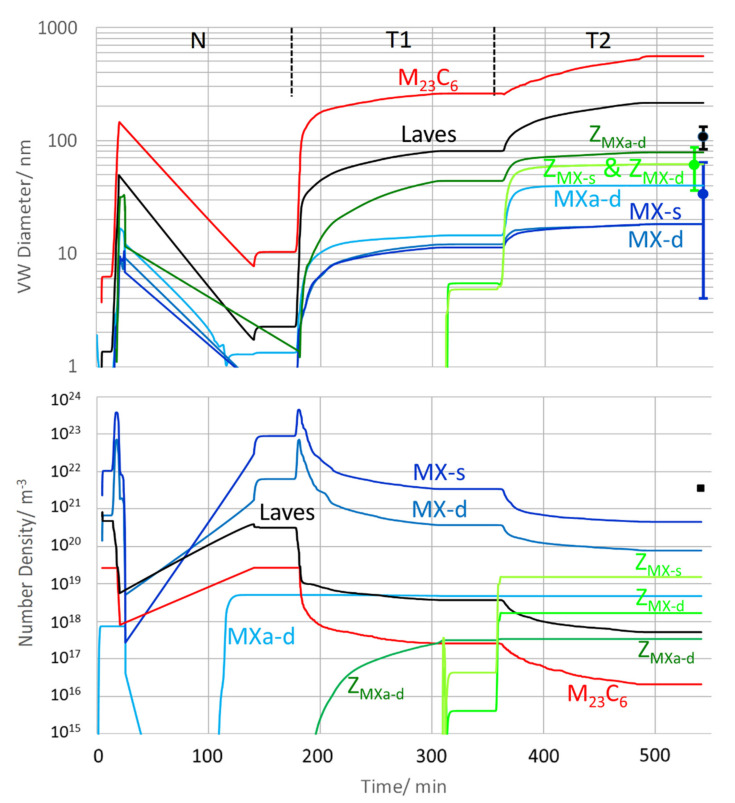
Simulated volume-weighted (VW) diameters and number densities during N + T of Z6 vs. TEM data from Table 5.

Figure 10 summarizes the simulated evolution of mean diameters and number densities of all precipitates during normalizing (N), tempering step 1 at 650 °C (T1) and tempering step 2 at 700 °C (T2). In condition 1, the TEM data from this work (from Table 5 in Section 5.1) are included.

The nucleation of MX precursors is started during normalizing, whereas transformation into Z-phase first takes place during tempering step T1. MX nucleating at subgrain boundaries (“*MX-s*”) and at dislocations (“*MX-d*”) in martensite reach diameters of 18 nm after T2, which is in satisfactory agreement with the TEM results. The modelled MX originating from dislocations in austenite (“*MXa-d*”) fit well to the measurements with a diameter of 40 nm. Z-phases transforming from the two smaller MX variants (“*Z*_MX-s_” and “*Z*_MX-d_") developed a mean size of 62 nm, showing excellent agreement with our TEM data. The Z-phase forming at the bigger MX variant (“*Z*_MXa-d_”) had a size of 78 nm. The simulated Laves phase diameter of 215 nm was overestimated compared to the measured value of 108 nm. However, the number of evaluated particles in TEM was very low; it should be noted that the Laves phase documented in [18] for a similar steel was in the range of 200 nm. M_23_C_6_ become very large in the simulation with around 541 nm. Their size has not been experimentally determined in the N+T stage.

Number densities of “*MX-s*” and “*MX-d*” have their maximum after normalizing, before a continuous reduction occurs during tempering. Both simulation and TEM measurements confirm that after T2, MX remains the most frequent particle type, followed by Z-phase. The overall number density lies between 5 × 10^20^ m^−3^ (simulated) and 3.7 × 10^21^ m^−3^ (measured). For evaluation of the experimental number density, area densities were com­bined with diameters and the sample size (approximately 115 nm) according to method 3 from [54].

**Figure 11:**
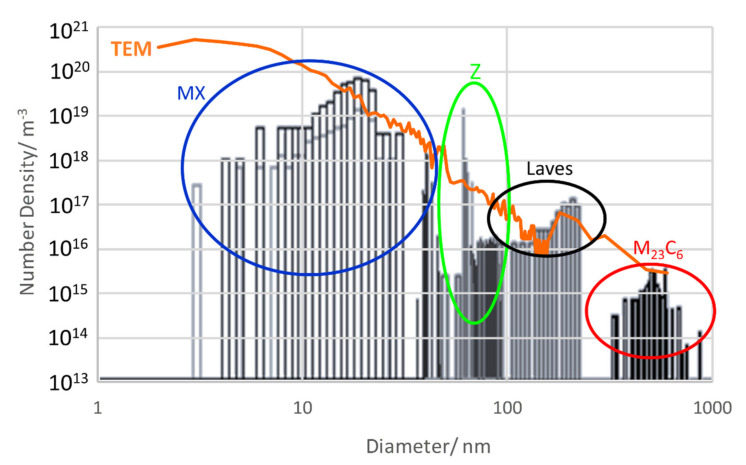
Size distributions of Z6 in as-received condition 1 (N + T); simulated vs. measured.

For a deeper insight into the difference of number densities between the simulation and the TEM measurement, it is worth taking a closer look at size distributions. Figure 11 gives us the opportunity to do so in as-received condition 1 (N + T). The black columns represent size classes from the thermokinetic simulation, and the coloured circles mark to which modelled precipitate population they belong. In comparison, the full orange-coloured line is derived from analysis of TEM data with the same method [54]. For a discussion of the size distributions, please refer to Section 7.6.

#### 6.3.2. Z6, Ageing Up to Condition 2

**Figure 12:**
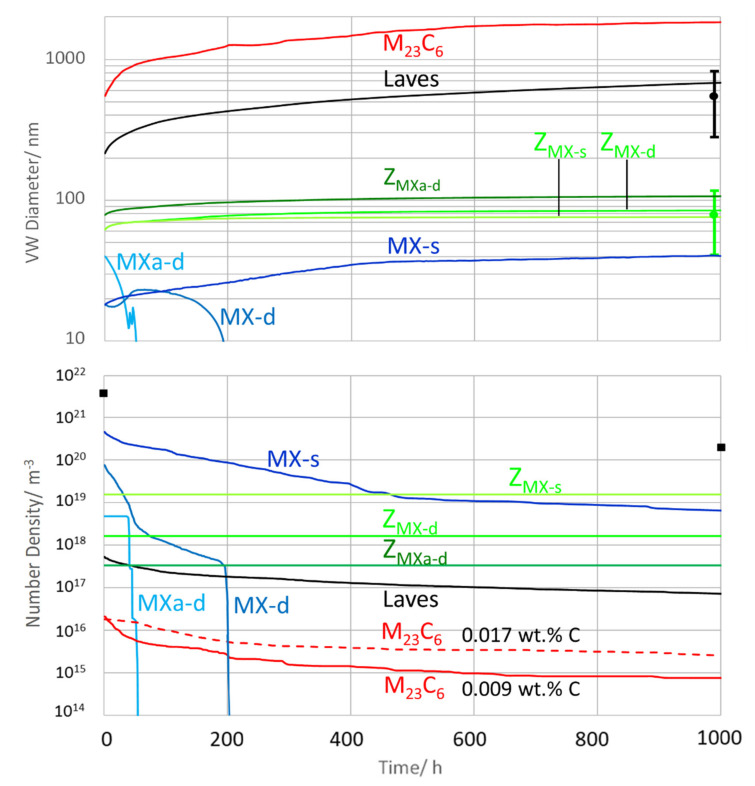
Simulated volume-weighted (VW) diameters and number densities during ageing of Z6 vs. TEM data from Table 5.

Figure 12 summarizes all changes of mean sizes and number densities of precipitates during ageing at 700 °C for 1000 h.

**Figure 13:**
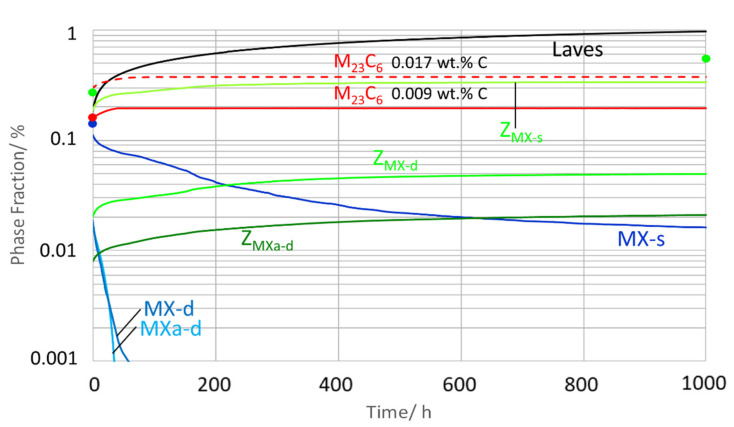
Simulated phase fractions during ageing of Z6 at 700 °C (condition 1 up to condition 2).

During ageing, all ongoing transformations of MX into Z-phases are completed. The two most prominent Z-phases “*Z*_MX-s_” and “*Z*_MX-d_” coarsened to sizes of 76 and 84 nm, which is in excellent agreement with the TEM measurements. “*Z*_MXa-d_” reached 107 nm and covered the upper end of the measured TEM scatter band. “*MXa-d*” and “*MX-d*” disappeared after 60 and 80 hours, respectively, and were fully replaced by the corresponding Z-phases. Only tiny quantities of “*MX-s*” remain stable without transition into Z-phase (around 0.016% phase fraction; see Figure 13), reaching a size of 40 nm. Laves phase coarsens to 682 nm and M_23_C_6_ to 1835 nm. Laves phase was in good line with evaluated TEM data, and M_23_C_6_ proved to be in a reasonable range, since the most observed grain boundary carbides in the aged condition were in the range of 1 to 3 µm.

In terms of number density, “*Z*_MX-s_” maintained the highest value after 1000 h of ageing at 700 °C at around 2 × 10^19^ m^−3^. “*MX-s*” and “*Z*_MX-d_” followed at approximately 7 × 10^18^ m^−3^ and 2 × 10^18^ m^−3^. It was confirmed by TEM that the overall number density during ageing at 700 °C strongly decreased and that Z-phase became the most frequent particle type. In addition to the simulated M_23_C_6_ result with 0.009 wt.% C, the diagram also includes the M_23_C_6_ result with 0.017 wt.% C. Again, we note a discrepancy between simulation (3 × 10^19^ m^−3^) and TEM data (2 × 10^20^ m^−3^) of the overall number density.

Figure 13 displays the evolution of the molar phase fractions during ageing.

Laves phase reached the highest molar phase fraction with about 0.97% in condition 2. Simulated Z-phase “*Z*_MX-s_” evolved from 0.19% in condition 1 to 0.34% in condition 2. In condition 2, “*Z*_MX-d_” and “*Z*_MX-a-d_” contributed another 0.07%. In total, the calculated fraction of all Z-phases increased from 0.21% in condition 1 to 0.41% in condition 2. This result is in good agreement with the evaluation of our TEM data according to method 3 in [54], yielding 0.27% in condition 1 and 0.55% in condition 2. The simulated MX phase fraction decreased from 0.12% in condition 1 (which fits well with a measured quantity of 0.14%) to 0.02% in condition 2. Simulated M_23_C_6_ phase fraction in condition 1 was 0.17 to 0.31% and in condition 2 lay between 0.20 and 0.34% (for 0.009 for 0.017 wt.% C). This fits well with the measured quantity of 0.16% in condition 1. (Due to the large diameter of M_23_C_6_ in condition 2, statistics for a systematic evaluation of the number density by TEM were not sufficient.)

**Figure 14:**
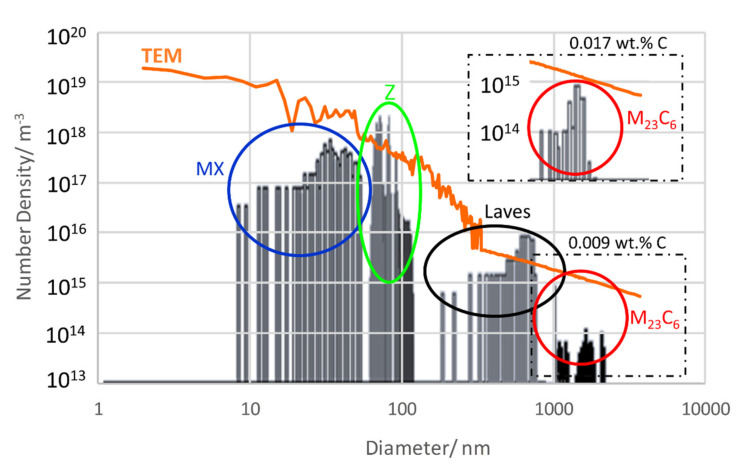
Size distributions of Z6 in aged condition 2 (700 °C/1000 h); simulated vs. measured.

Once more, we aim to clarify the origin of the difference between simulated and measured number densities (orange line) from investigating size distributions in condition 2; see Figure 14. The diagram shows a better agreement for M_23_C_6_, when selecting 0.017 wt.% C for simulation instead of 0.009 wt.% C.

## 7. Discussion

### 7.1. Z-Phase Precursors MX

S, Ti, V, Cr, Fe, Nb, Mo, Ta and W were considered within the TEM-EDX measurements; however, the standard deviation of the S, Ti, Nb and Mo signals significantly exceeded the signal dimension itself. Therefore, these elements are excluded from further discussion.

Table 8 compares the simulated chemical composition of MX to our TEM-EDX data. In addition, APT results of Z-steels from the literature are shown, identifying two different chemistries for MX. Type A is almost free of C, whereas type B contains a considerable amount of C.

When normalized to the metallic part of MX, the MatCalc result (80 min N) fits well with the results of TEM-EDX (as-received) and APT of particle type B (as-received). The MatCalc result (as-received) fits well with the APT of particle type A (as-received). It appears there is competition between Ta-rich carbonitrides and Cr-rich nitrides of type MX.

**Figure 15:**
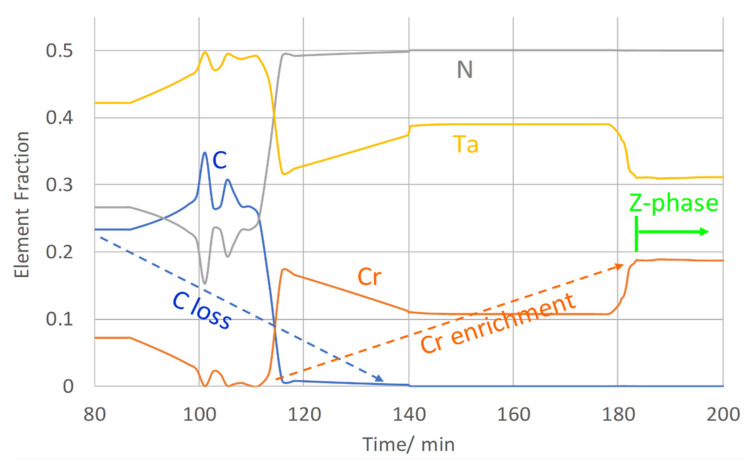
Outtakes of MX chemistry evolution during normalizing and tempering T1.

In order to discuss this situation in more detail, we analyze the evolution of the chemical composition of MX precipitates during normalizing N and tempering T1 up to the start of Z-phase transformation (see Figure 15).

During normalizing, MX first forms as Ta-rich carbonitride. In the further course of the heat treatment, the precipitates enrich in both N and Cr. Apparently, this process can be faster or slower for individual particles—see the distinction between particle types A and B in [18,22]. As soon as the Cr-content reaches a critical level, the transformation of MX into the Z-phase starts, as indicated by the green arrow in Figure 15.

### 7.2. Z-Phase Precursors M_2_X

Theoretically, M_2_X precursors may also act as spots for Z-phase (trans-)formation, which is in this case accompanied by Ta intake. However, M_2_X precursors were not selected for Z6 in the thermokinetics simulation since they were not found experimentally. The low quantity of excess N (corresponding to a ratio of Ta:N ≈ 1) probably favours nucleation of MX instead of M_2_X [22]. Small quantities of M_2_X do appear in the equilibrium calculations (0.02 to 0.03% phase fraction) but have very little affinity for Ta. In fact, they are Cr_2_N with only traces of Ta (<0.2 at.%).

### 7.3. Z-Phase Size Evolution

The simulated evolution of Z-phase size shows excellent agreement with TEM measurements. An important factor to enable a realistic coarsening rate was permitting Cr to also occupy the second sublattice in the thermodynamic description. This means that Cr continues to diffuse into the Z-phase until reaching the equilibrium condition of Cr_1.2_Ta_0.8_N [24]. When simplifying Z-phase to a compound CrTaN and locking out excess Cr, the coarsening is remarkably less pronounced, producing an error of around 25% with respect to the Z-phase size after 1000 h of ageing at 700 °C. Moreover, when using the simpler sublattice version, triple the phase fraction of MX remains stable without trans­formation after 1000 h of ageing (0.06%) when compared to offering a Cr_1.2_Ta_0.8_N sublattice model (0.02%). These findings indicate the relevance of appropriate and complete site occupancies for a correct prediction of the MX transformation into the Z-phase.

### 7.4. Chemical Composition of Z-Phase Precipitates

S, Ti, V, Cr, Fe, Nb, Mo, Ta and W were considered within the TEM-EDX measurements; however, the standard deviation of the S, Ti, Mo and Nb signal significantly exceeded the size of the signal. Therefore, these elements are excluded from further discussion.

Simulated chemical compositions of Z-phases fit very well with APT results from the literature and with our TEM-EDX results, as shown by the comparison between MatCalc and experimental data in Table 9.

Although the ageing temperatures were different (700 °C in our case, 650 °C in the literature), it is evident that the same equilibrium stoichiometry of Cr_1.2_Ta_0.8_N is reached. Hence, the replacement of the original (Cr)(Ta)(N) sublattice description in our database by a more complex (Cr)(Cr,Ta)(N) system for the Z-phase turned out to be successful.

Agreement of the simulation results with our TEM-EDX data is also very good. Even though enrichment of Z-phase with V seems to happen earlier in reality than in simulation, both measurement and MatCalc claim 2 at.% of V to enter.

### 7.5. Calibration of Transformation Model

In the thermokinetic simulation setup, the minimum nucleation radius *mnr* can be understood as the smallest radius of a precipitate that represents the crystal structure of the corresponding phase. For the modified Z-phase, *mnr* was estimated by investigations of the elementary cell, yielding lattice parameters of a = 2.86–3 Å and c = 7.39 Å [16,53]. Anything smaller than approximately 5 Å is therefore an atomic cluster rather than an ordered crystallite, thus making our choice for this *mnr* a reasonable one.

While our Z-phase transformation model exhibits excellent results compared to the experimental data—both in terms of chemical composition and also for the size evolution-, the use of a fitted equivalent interface energy $\gamma_{0,\mathrm{equ}}$ undoubtedly reveals a weakness of the model. Although the model uses a phenomenological twist to depict a real physical phenomenon, it allows us to bridge metastable MX to stable Z-phase condition. In this context, $\gamma_{0,\mathrm{equ}}$ controls the transformation speed. The higher $\gamma_{0,\mathrm{equ}}$ is chosen, the slower the transition is from MX to Z-phase that will be realized. Despite its unknown exact value, the range of possible energies for Z-phase seems to lie between 0.1 and 0.4 Jm^−2^, as ongoing MatCalc simulations for similar materials involving CrVN Z-phase have shown. Whereas in Z6, $\gamma_{0,\mathrm{equ}}$ was selected as 0.22 Jm^−2^, it had to be chosen higher in, e.g., P91 (with 0.39 Jm^−2^) due to slower CrVN Z-phase formation.

### 7.6. Overall Number Densities and Size Distributions

The simulated number densities differ from our TEM data by around one order of magnitude. During ageing, the total number density decreased from around 10^21^ m^−3^ in condition 1 to 10^20^ m^−3^ in condition 2 according to TEM. By contrast, the simulation predicts a reduction from 10^20^ m^−3^ in condition 1 to 10^19^ m^−3^ in condition 2. Whereas in as-received condition, MX are most frequent, it is the Z-phase that becomes most prominent after ageing; this is confirmed both by measurement and simulation.

**Figure 16:**
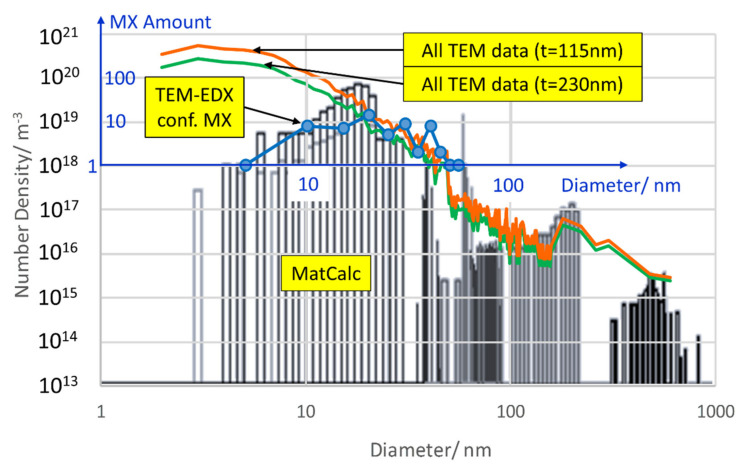
Size distributions of Z6 in condition. 1 (N + T); simulated vs. measured—all data and TEM-EDX confirmed MX sizes within the standard deviation.

In order to get a more detailed view on the agreement of simulated and measured number densities, we take a closer look at the corresponding size distributions; see Figure 14 and Figure 16.

Apparently, the agreement is very good for precipitates larger than 10 nm, whereas substantially more small particles were identified in the TEM replica compared to the sim­ulation results. Either the extraction replicas produce artefacts (see e.g., [55]) due to a detection limit for small particles or the simulation underestimates the number of small particles. When excluding precipitates <10 nm in condition 1 from the measured data, the number density would decrease remarkably to 8 × 10^20^ m^−3^ and move closer to the simulation result of 5 × 10^20^ m^−3^ or reduce to 9 × 10^19^ m^−3^ as compared to a simulated number of 3 × 10^19^ m^−3^ in condition 2. However, since the detection limit for confirming the nature of particles by EDX is about 10 nm, the question of whether results from TEM or the simulation are closer to reality has to be left open at this point.

One reason for the deviation between simulated and measured number densities is the sample thickness of the TEM samples. For calculating the measured number densities, we assumed a mean thickness of 115 nm. However, TEM sample thickness is not homogeneous and can differ locally. According to our evaluation method, doubling the assumed thickness for the same micrograph leads to about half the number of particles per volume for the smallest precipitates; see green line in Figure 16. For large precipitates (e.g., >100 nm) this effect is less pronounced, and only 20 to 30% number density reduction can be observed. Thus, the sample thickness can be part of the reason for the deviances.

Another possible source for the deviation is the mean-field approximation made in the simulation approach. Although the experimental size distributions of precipitates often show right-skewed log-normal shapes, the mean-field approximation delivers left-skewed distributions. This issue has been discussed recently by de Nunzio [56,57] and will not be further elaborated here.

### 7.7. C Content and Amount of M_23_C_6_

C content has been previously determined on a sample taken from the melt liquid by combustion analysis, revealing only 0.009 wt.% of C [29]. The notable discrepancy to 0.017 wt.% determined by OEM in this work might be related to segregation effects during solidification, respectively, to an inhomogeneous distribution of C. This difference first of all impacts the M_23_C_6_ quantity. Equilibrium test calculations (see chapter 6.1) led to a molar phase fraction of only 0.2% of M_23_C_6_ with 0.009 wt.% C, whereas we obtained 0.4% M_23_C_6_ with 0.017 wt.% C. Evaluation of size distributions in condition 2 give a hint that the C-content of 0.017 wt.% detected by OEM seems to be more representative for the zones investigated by TEM.

### 7.8. BN Implications

Equation (4) describes the solubility limit of BN at 1150 °C in a 9% Cr-steel [58].

(4) $$\log\left(wt.\%B\right)=-2.45\cdot \log\left(wt.\%N\right)-6.81$$

Evaluation shows only 0.019 out of 0.035 wt.% N to be soluble in the matrix of Z6. This speaks for the formation of BN in Z6. However, no BN was detected in similarly alloyed ZULC [18]. A reason for this might be that Cr decreases the activity of N [59], with the result that BN formation is more difficult in 12% Cr steels compared to 9% Cr steels [18]. Apart from this, the C content—which is argued to raise the activity of B [18]—is much lower in Z6 and in ZULC compared to alloys investigated by [58]. Also in Z6, BN was not found by TEM. The MatCalc equilibrium calculation for Z6 predicts a small molar phase fraction of 0.03% BN. With a documented size in the µm range [18,22], a number density in reality of approximately 10^14^ m^−3^ can be estimated. With this number density, it is extremely unlikely that these particles are found in TEM. Furthermore, large BNs are known to be very stable after formation. For this reason, we decided to consider BN by calculating an effective composition of the material, reduced by the amount of N bound by the BN particles (0.031–0.035 wt.%). Thus, we correctly took into account the impact of BN on MX formation.

## 8. Conclusions

- Element Ta and its interactions with Cr, N and C—determining the nucleation behaviour of MX precursors and enabling Z-phase to form—have been successfully implemented into the thermodynamic steel database *mc_fe*.
- The dissolution temperature of Ta-based Z-phase contributed to the parametrization of the thermodynamic model.
- A model based on inner-particle nucleation was applied for the transformation of metastable MX precursors into Z-phase controlled by Cr intake.
- The parameter setup for the thermokinetic calculation involves detailed micro­structural input data—in particular dislocation densities—which were gained from TEM measurements and combined with modelling of dislocation evolution.
- The simulation results were validated based on our TEM precipitate results as well as APT data from the literature. Modelled Z-phase size, number density and chemical composition showed excellent agreement to measurements. The simulation greatly contributed to the interpretation of the experimental results from TEM analysis (especially to the size distributions).
- Thermokinetic simulation tools as presented here can assist improved engineering of novel creep-resistant materials to make thermal power plant operation safer, more predictable and more efficient.

## 9. Outlook

Decoding the correct interaction of V with Cr, Ta and N in MX remains a task for the future. To the knowledge of the authors, at the moment, no experimental data are available on phase stabilities and solid solutions in the system Cr-Ta-V-N.

Implementation of the presented microstructural and phase data into a recently pub­lished mean-field dislocation creep model [60] is planned.

## Figures and Tables

**Table 1:** Chemical composition of materials in wt.%.

wt.%	Fe	Ni	Cu	Cr	W	Mo	Si
ZULC	bal	0.50	-	11.79	2.90	-	0.30
Z6	bal	0.22	0.98	10.90	1.70	0.74	0.05
**wt.%**	**Mn**	**C**	**N**	**Co**	**Ta**	**B**	**V**
ZULC	0.48	0.005	0.033	7.30	0.39	0.004	-
Z6	0.49	0.009–0.017	0.035	3.71	0.38	0.003	0.013

**Table 2:** Revised sublattice descriptions and thermodynamic interaction parameters with Ta (this work).

**BCC_A2**	: Co,Cr,Cu,Fe%,Mn,Mo,Ni,Si,Ta,V,W : B,C,N,Va% :
Interaction Parameters	G0Ta:NBCC_A2=5·104+G0TaSER+3·G0NSER
	L0Ta:N,VaBCC_A2=125·104−280·T
**FCC_A1**	: Co,Cr,Cu,Fe%,Mn,Mo,Ni,Si,Ta,V,W : B,C,N,Va% :
Interaction Parameters	G0Ta:CFCC_A1=G0TaSER+3·G0CSER−5·104−60·T
	G0Ta:NFCC_A1=G0TaSER+3·G0NSER−19·104+95·T
	L0Ta:N,VaBCC_A2=10−8
	L0Cr,Ta:NBCC_A2=−55·103
**HCP_A3**	: Co,Cr,Cu,Fe%,Mn,Mo,Ni,Si,Ta,V,W : B,C,N,Va% :
Interaction Parameters	G0Ta:CHCP_A3=G0TaSER+0.5·G0CSER−105+15·T
	G0Ta:NHCP_A3=G0TaSER+0.5·G0NSER−75·103+30·T
	L0Cr,Ta:VaHCP_A3=−25·103
	L0Cr,Ta:NHCP_A3=−25·103
	L0Ta:N,VaHCP_A3=−25·103
**Z-Phase**	: Cr%,Fe : Cr,Nb,Mo,Ta,V : N%,Va :
Interaction Parameters	G0Cr:Cr:NZ−phase=G0CrSER+G0CrSER+G0NSER+15·103
	G0Cr:Cr:VaZ−phase=G0CrSER+G0CrSER+2·104
	G0Cr:Ta:NZ−phase=−225 ·103+102·T+G0CrSER+G0TaSER+G0NSER
	G0Cr:Ta:VaZ−phase=G0CrSER+G0TaSER
	L0Cr:Ta:N,VaZ−phase=−2·105+85·T
	G0Cr:Cr,Ta:NZ−phase=−11·104+18·T

The code of all new interaction parameters is available herein as supplement part 1.

**Table 3:** Z6 nucleation sites for precipitate kinetic simulation.

Nucleation Sites Z6		MX	M_23_C_6_	Laves	Z
Matrix	Austenite	d	-	-	-
	Martensite	d, s	s	g	MX_i_
g = Grain Boundaries; d = Dislocations; s = Subgrain Boundaries; MX_i_ = MX as Z-phase Nucleation Sites (On-Particle Nucleation)					

**Table 4:** Input parameters for MatCalc 6.03 precipitate kinetic simulation of Z6.

Input MatCalc		Value		Source
Databases		mc_fe_v2.061.tdb and mc_fe_v2.013.ddb		This work
Heat Treatment	Normalizing	1 h @ 1100 °C		[29]
	Tempering	2 h @ 650 °C & 2 h @ 750 °C		
	Ageing	1000 h @ 700 °C		
PAGS		48 µm		[29]
Subgrain Size		0.41 µm		TEM
Dislocation Density	Austenite	1 × 10^11^ m^−2^		[45]
	Fresh Martensite	6.0 × 10^14^ m^−2^	Equation (3) *C* = 3 × 10^−4^	[50] calibrated by TEM (this work)
	Tempered Martensite	2.6 × 10^14^ m^−2^		
	Ageing	Start: 2.6 × 10^14^ m^−2^ End: 8 × 10^13^ m^−2^		
Martensite Start Temperature		420 °C		[29]
Reaustenitization Temperature		820 °C		[29]
Z-Phase	$\gamma_{0,\mathrm{equ}}$	0.22 Jm^−2^		Fit
	*mnr*	5 Å		[16,53]

**Table 5:** Precipitate sizes and number densities from Z6 TEM analysis.

Cond.	Prec.	*D*_i_/nm	*N*_EDX_	*N*_V_/m^−3^	*N*_Size_
1	MX	34 ± 30	64	3.7 × 10^21^	4246
	Z	61 ± 25	34		
	Laves	108 ± 24	5		
2	Z	79 ± 38	88	2 × 10^20^	2434
	Laves	551 ± 270	32		
	M_23_C_6_	2728 ± 954	6		

**Table 6:** Dislocation densities and subgrain sizes from Z6 TEM analysis.

Cond.	Microstructure	*ρ*_int_ [m^−2^]	*D*_sgb_ [µm]
0	Martensite	6.0 ± 0.5 × 10^14^	0.48 ± 0.09
1	Tempered Martens.	2.6 ± 0.6 × 10^14^	0.41 ± 0.18
2	Aged Temp. Martens.	7.9 ± 2.1 × 10^13^	0.43 ± 0.14

**Table 7:** Calculated equilibrium phase fraction in mol.% for ZULC and Z6.

Material	Precipitate	550 °C	600 °C	650 °C	700 °C
		[mol.%]	[mol.%]	[mol.%]	[mol.%]
ZULC	Laves	2.54	2.43	2.26	1.99
	Z-phase	0.41	0.41	0.41	0.41
	M_23_C_6_	0.12	0.11	0.11	0.11
	BN	0.04	0.04	0.04	0.04
Z6	Laves	2.36	2.10	1.71	1.16
	Z-phase	0.42	0.42	0.42	0.41
	M_23_C_6_	0.20–0.39	0.20–0.39	0.20–0.38	0.20–0.38
	MX (fcc)	None	0.02	0.02	0.02
	Cr_2_N (hcp)	0.02	None	None	None
	BN	0.03	0.03	0.03	0.03

**Table 8:** MX chemistry of 80 min N + cond. 1 in MatCalc (this work), TEM-EDX (this work) and APT (literature).

MX Simulation Z6 (**MatCalc**)						MX Literature (**APT**)						
Cond.	Chem. Comp. [at.%]					Cond.	Chem. Comp. [at.%]				Type	Ref.
	Cr	N	Ta	V	C		Cr	N	Ta	C		
80 min N	7.2	26.6	42.2	0.0	23.3	As-rec.	42.6	45.9	6.6	0.4	A	[18]
							35.8	48.1	9.3	0.8	A	[22]
As-rec. (N + T)	26.7	50.0	22.9	0.3	0.0		17.2	13.8	27.1	32.6	B	[18]
							18.4	29.4	27.0	13.3	B	[22]
MX Measured (**TEM-EDX**)												
Cond.	Chem. Comp. [at.%]											
	Cr	Fe	Ta	V	W							
As-rec.	14 ± 5	3 ± 2	75 ± 9	<1	5 ± 3							

**Table 9:** Z-ph. chemistry of cond. 1 + 2 in MatCalc (this work), TEM-EDX (this work) and APT (literature).

Z-Phase Simulation Z6 (**MatCalc**)						Z-Phase Literature (**APT**)				
Cond.	Chem. Comp. [at.%]					Cond.	Chem. Comp. [at.%]			Ref.
	Cr	N	Ta	V			Cr	N	Ta	
As-rec.	36.9	27.3	35.7	0.1		As-rec.	33.6	26.9	30.2	[18]
10^3^ h/700 °C	40.7	28.7	28.4	2.2		10^4^ h/650 °C	39.8	30.2	24.1	[18]
Z-Phase Measured (**TEM-EDX**)										
Cond.	Chem. Comp. [at.%]									
	Cr	Fe	Ta	V	W					
As-rec.	51 ± 7	6 ± 2	37 ± 8	2 ± 1	3 ± 2					
10^3^ h/700 °C	52 ± 4	5 ± 2	36 ± 3	2 ± 1	3 ± 1					

## Data Availability

The code of the thermodynamic database is provided as Supplement part 1 (Word). Obtained MatCalc simulation data are contained in Supplement part 2 (Excel).

## Supplementary Materials

The following are available online at https://www.mdpi.com/1996-1944/14/6/1332/s1: Part 1 (Word file): code of the thermodynamic database; Part 2 (Excel file): MatCalc simulation data.
