# Supplementary material for: Thermodynamic Modelling and Microstructural Study of Z-Phase Formation in a Ta-Alloyed Martensitic Steel
Source: Materials (Basel). 2021 Mar 10;14(6):1332. doi: 10.3390/ma14061332 (PMC7998842; doi:10.3390/ma14061332)
Supplement: Supplementary file 1 [file materials-14-01332-s001.zip › Supplement-Part1.docx]

**Supplementary material for**:

F. Riedlsperger, B. Gsellmann, E. Povoden-Karadeniz, O. Tassa, S. Matera, M. Domankova, F. Kauffmann, E. Kozeschnik and B. Sonderegger, “*Thermodynamic modelling and microstructural study of Z-phase formation in a Ta-alloy martensitic steel* ”, MDPI Materials 2021, 14, xx

*Part 1 (this document):* Thermodynamic parameters and diffusion mobilities to be included in open source steel databases“mc_fe.tdb” and “mc_fe.ddb”, see online: <https://www.matcalc.at/index.php/databases/open-databases>

*Part 2 (Excel file):* MatCalc simulation data (equilibrium results of ZULC and Z6 as well as precipitate kinetic results of Z6)

**Part 1:**

1. Thermodynamic parameters:

$ Definition of element
ELEMENT TA BCC_A2 80.9479 5681.872 41.4718 !

$ MatCalc specific commands
ADD_COMPOSITION_SET FCC_A1 :TA,TI,NB,V:C,N: !
ADD_COMPOSITION_SET HCP_A3 :CR,TA:N: !
ADD_COMPOSITION_SET HCP_A3 :MO:C: !
ADD_COMPOSITION_SET HCP_A3 :CR,V:C: !

$ Phase descriptions
PHASE BCC_A2 %& 2 1 3 !
 CONSTITUENT BCC_A2 : CO,CR,CU,FE%,MN,MO,NI,SI,TA,V,W :B,C,N,VA% : !
 PARAMETER G(BCC_A2,TA:N;0) 273.00 +500000+GHSERTA#+3*GHSERNN#; 6000.00 N
 PARAMETER L(BCC_A2,TA:N,VA;1) 273.00 +1250000-280*T; 6000.00 N

PHASE FCC_A1 %' 2 1 1 !
 CONSTITUENT FCC_A1 : CO,CR,CU,FE%,MN,MO,NI,SI,TA,V,W :B,C,N,VA% : !
 PARAMETER G(FCC_A1,TA:C;0) 273.00 +GHSERTA#+GHSERCC#-50000-60*T; 6000.00 N
 PARAMETER G(FCC_A1,TA:N;0) 273.00 +GHSERTA#+GHSERNN#-190000+95*T; 6000.00 N
 PARAMETER L(FCC_A1,TA:N,VA;0) 273.00 +1e-8; 6000.00 N
 PARAMETER L(FCC_A1,CR,TA:N;0) 273.00 -55000; 6000.00 N

PHASE HCP_A3 %) 2 1 0.5 !
 CONSTITUENT HCP_A3 : CO,CR,CU,FE%,MN,MO,NI,SI,TA,V,W :B,C,N,VA% : !
 PARAMETER G(HCP_A3,TA:C;0) 273.00 +GHSERTA#+0.5*GHSERCC#-100000+15*T;
6000.00 N
 PARAMETER L(HCP_A3,CR,TA:VA;0) 273.00 -25000; 6000.00 N
 PARAMETER G(HCP_A3,TA:N;0) 273.00 +1*GHSERTA#+0.5*GHSERNN#-75000+30*T;
6000.00 N
PARAMETER L(HCP_A3,CR,TA:N;0) 273.00 -25000; 6000.00 N
PARAMETER L(HCP_A3,TA:N,VA;0) 273.00 -25000; 6000.00 N

PHASE ZET % 3 1 1 1 !
 CONSTITUENT ZET : CR%,FE : CR,NB,MO,TA,V : N%,VA : !
 PARAMETER G(ZET,CR:CR:N;0) 273.00 +GHSERCR#+GHSERCR#+GHSERNN#+15000; 6000.00 N
 PARAMETER G(ZET,CR:CR:VA;0) 273.00 +GHSERCR#+GHSERCR#+20000; 6000.00 N
 PARAMETER G(ZET,CR:TA:N;0) 273.00 -25000+102*T+GHSERCR#+GHSERTA#+GHSERNN#; 6000.00 N
 PARAMETER G(ZET,CR:TA:VA;0) 273.00 +GHSERCR#+GHSERTA#; 6000.00 N
 PARAMETER L(ZET,CR:TA:N,VA;0) 273.00 -200000+85*T; 6000.00 N
 PARAMETER G(ZET,CR:CR,TA:N;0) 273.00 -110000+18*T; 6000.00 N

1. Diffusion mobilities:

PARAMETER MQ(FCC_A1&TA,*) 273.00 -QFE_FCC+R*T*LN(5.0*D0FE_FCC); 6000.00 N !
PARAMETER MQ(BCC_A2&TA,*) 273.00 -QFE_BCC; 6000.00 N
PARAMETER MF(BCC_A2&TA,*) 273.00 +R*T*LN(3.0*D0FE_BCC); 6000.00 N
